# Implementation of a crossed-slit system for fast alignment of sealed polycapillary X-ray optics

**DOI:** 10.1107/S1600577520012217

**Published:** 2020-10-26

**Authors:** Anna Zymaková, Krishna Khakurel, Alessandra Picchiotti, Wojciech Błachucki, Jakub Szlachetko, Mateusz Rebarz, Jens Uhlig, Jakob Andreasson

**Affiliations:** a RP4, ELI Beamlines, Za Radnici 835, Dolni Brezany 25241, Czech Republic; b Institute of Nuclear Physics, Polish Academy of Sciences, Radzikowskiego 152, Krakow 31342, Poland; c Division of Chemical Physics, Lund University, Box 117, Lund 22100, Sweden

**Keywords:** X-ray focusing optics, polycapillaries, X-ray sources, alignment, X-ray slits

## Abstract

A new modification of a table-top laser-driven water-jet plasma X-ray source has been successfully implemented and commissioned at the Extreme Light Infrastructure (ELI) Beamlines user facility. In order to preserve the broadband nature of the source for spectroscopic experiments, a polycapillary lens was initially chosen as the focusing element. Generally, polycapillary X-ray optics have a narrow photon acceptance angle and small field of view, making alignment complicated and time-consuming. This contribution demonstrates a straightforward, reliable and reproducible procedure for aligning polycapillary focusing optics with broadband X-rays. The method involves a pre-alignment step where two X-ray slits are mounted orthogonally on opposite sides of a 3D-printed cylindrical polycapillary holder. This helps to precisely determine the optical axis of the X-ray beam. Subsequent mounting of the polycapillary in the pre-aligned holder with the slits removed allowed for immediate transmission of the X-ray photons through the optics and has provided a good starting point for fine alignment.

## Abstract 2

A fast X-ray focusing polycapillary alignment method was implemented and tested using a compact short-pulse laser-driven broadband X-ray source.

## 1. Introduction   

The advent of compact table-top laser-driven pulsed X-ray sources (Korn *et al.*, 2002 ▸; Zamponi *et al.*, 2009 ▸; Uhlig *et al.*, 2013 ▸) has enabled significant progress in time-resolved studies (Rischel *et al.*, 1997 ▸; Rousse *et al.*, 2001 ▸; Hauf *et al.*, 2019 ▸; Lu *et al.*, 2020 ▸) using mid- to high-power laser systems (*e.g.* Batysta *et al.*, 2016 ▸). Such table-top sources offer researchers easier access to pulsed X-ray radiation, and important advantages such as improved temporal resolution compared with synchrotrons (Iqbal *et al.*, 2014 ▸) and inherent synchronization between pump and probe beams. However, even the sources driven by state-of-the-art lasers have limitations in photon flux. Previous reports suggest that, depending on the source type and driving laser parameters, one can expect a photon flux on the order of several 10^9^ photons sterad^−1^ s^−1^ (Weiss­haupt *et al.*, 2014 ▸; Iqbal *et al.*, 2014 ▸) at ∼8 keV up to 10^10^ photons sterad^−1^ s^−1^ at ∼4.5 keV (Afshari *et al.*, 2020 ▸). While the total flux is comparable with the flux from second-generation large-scale facilities, the radiation is emitted into 4π. The flux effectively used in experiments is significantly lower. Furthermore, in pump–probe experiments the spot size on the sample is severely limited by the accessible laser pump intensity and geometrical constraints to reduce temporal uncertainty. Thus, the inclusion of focusing X-ray optics is highly relevant (Bargheer *et al.*, 2005 ▸).

Developments in X-ray science have largely been facilitated by technological advancements in X-ray optics (Dabagov & Gladkikh, 2019 ▸). Each optics offers its own set of advantages but also suffers from characteristic limitations. Owing to its tolerance to broadband X-rays, in our setup we initially chose a polycapillary lens for re-imaging of the source spot onto the target. The origin of the polycapillary lens dates back to the 1990s (Kumakhov, 1990 ▸). A polycapillary consists of a number of curved channels, each guiding a photon to a required target spot, which is the focus. The tapered shape of a capillary enhances the exit radiation power (Cappuccio & Dabagov, 2000 ▸), or, if optics are flipped, defocuses it to, for example, a quasiparallel beam. The photon incident angle on a capillary wall must be smaller than the Fresnel critical angle (for X-rays in the range 7 keV ≫ 1°) (Hirsch & Kellar, 1951 ▸). Therefore, the acceptance angle of a bunch of such capillaries (constituting a polycapillary) is quite narrow. Subsequently, it is already challenging to achieve some transmission of X-rays through the optics, and the polycapillary must be positioned carefully with respect to a spot-like X-ray source (Dabagov, 2003 ▸), often blindly with a precision of several tens of micrometres in three dimensions and a few degrees in two angles. In this communication, we demonstrate a reliable approach to achieve time-efficient and precise alignment.

## 2. Experimental details and alignment procedure   

The method of polycapillary alignment presented here was implemented and verified during the commissioning of a 4π laser-driven water-jet plasma X-ray source (PXS) (Uhlig *et al.*, 2013 ▸) used in an X-ray spectroscopy endstation (Szlachetko *et al.*, 2012 ▸) at the ELI Beamlines (results to be published elsewhere) which exploits a polycapillary lens, a CCD camera, and sample delivery systems for solid and liquid samples (Picchiotti *et al.*, 2015 ▸).

**Figure 1:**
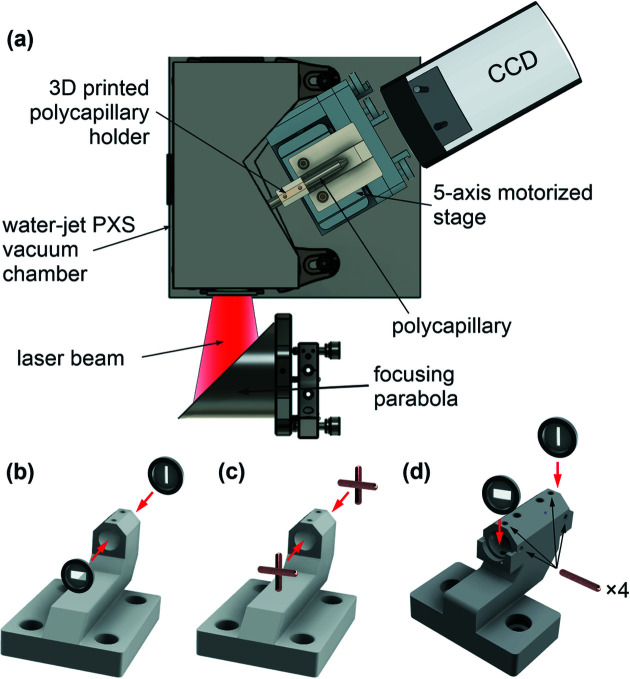
(*a*) Schematic of the experimental setup, (*b*) 3D-printed polycapillary holder pre-alignment with X-ray slits, (*c*) polycapillary holder pre-alignment with crossed wires and (*d*) upgraded version of the polycapillary holder: an alignment tube with pockets and holes for quick and precise slit/wire insertion/extraction.

In the present experiment, the driving laser was a Coherent Astrella amplifier with a central wavelength of 800 nm, repetition rate of 1 kHz, pulse duration of ∼40 fs and output pulse energy of 6.8 mJ. Approximately 4 mJ pulse energy was used to drive the water-jet PXS. The intensity of the source emission is a function of the laser pulse energy at a constant pulse duration (Miaja-Avila *et al.*, 2015 ▸). Thus, in our experiment at the energy of interest (∼7 keV, iron-based complexes), ∼100 photons (eV sterad shot)^−1^ could be expected. The experimental setup is shown schematically in Fig. 1 ▸(*a*).

The polycapillary optics (XOS) used in the experiment has 18 mm input and 30 mm output focal distance, an enclosure length of 77 mm and an effective entrance diameter of 3 mm. The output focal spot size (FWHM at 8 keV) is less than 130 µm. The polycapillary is sealed with thin beryllium windows on both sides. This makes pre-alignment with optical light (*i.e.* with the driving laser reflection from the water jet) impossible. The lengths of the central and the outermost capillaries are 73 mm and 73.19 mm, respectively. This path difference results in an expected stretching of the pulse duration by ∼0.6 ps. Hence, this optics is suitable for pump–probe experiments of ultrafast (on the order of picoseconds) dynamics. The entrance acceptance angle of the optics is approximately 8.6° if the source is perfectly positioned in a 50 µm × 50 µm input focal spot. However, each capillary has an acceptance angle of only ∼0.5°. If the point-like source is outside the focal spot, at most a single capillary accepts photons. Combined with the individual low transmission rate and limited source flux, alignment becomes a challenging and time-consuming task of defining five independent parameters (*x*, *y*, *z* and two rocking angles) with 50 µm per 0.5° precision, and a significant photon flux is transmitted only in cases of nearly perfect alignment.

Approaches using washers in order to find the optical axis of an X-ray beam have been applied in the work of Bingölbali & MacDonald (2009 ▸) and MacDonald (2010 ▸). Although a step in the right direction, this optical method lacks precision, thus necessitating further development of alignment procedures.

**Figure 2:**
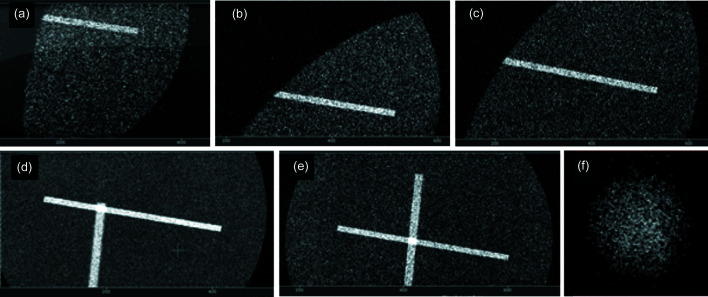
(*a*)–(*e*) Step-by-step process of polycapillary holder alignment using the perpendicular slits technique and (*f*) the result of focus fine alignment (enlarged for better visibility).

In our experiment, for easy alignment of the polycapillary lens, a holder was designed and 3D-printed in plastic [see Fig. 1 ▸(*b*)]. The holder has two threaded holes for rigid optics fixation and is mounted on a five-axis motorized stage (New Focus 8082M from Newport) for fine alignment of the lens. Prior to insertion of the polycapillary, an approximate path of the X-ray beam was determined, and the holder was roughly pre-aligned to the optical axis. At the entrance and exit of the holder, two X-ray slits, *i.e.* two round frames with long rectangular single openings, of 75 µm and 100 µm aperture/slit widths, were fixed perpendicularly to each other using double-sided adhesive tape [Fig. 1 ▸(*b*)]. The material of a slit frame should allow for partial X-ray transmission so that a clear picture formed by X-rays transmitted through the slits appears on the detector. A direct detection X-ray CCD was used for imaging of the X-ray photons. Fig. 2 ▸(*a*) shows the camera reading after the rough pre-alignment.

Next, the motors are moved to image both perpendicular slits and align the centers of the slits until a fine symmetric cross is observed. The gradual alignment process is shown in Figs. 2 ▸(*b*)–2(*e*). In Fig. 2 ▸(*e*) the central square of the cross has higher X-ray intensity as a result of photons transmitted through both slits while the cross arms remain darker. This procedure is followed by careful removal of the slits and mounting of the polycapillary lens in the holder. At this step, a faint X-ray spot is observed in the camera reading. The final step is fine tuning of the polycapillary with the five-axis motorized stage in order to obtain the brightest spot on the camera, according to the scanning procedure introduced in the work by Cappuccio & Dabagov (2000 ▸) and further improved by Cappuccio *et al.* (2001 ▸) and Cappuccio & Dabagov (2002 ▸), that is recognized as a standard technique for polycapillary optics alignment. As a result, an optimized image can be obtained as shown in Fig. 2 ▸(*f*).

The alignment procedure described in this communication requires the use of two X-ray slits. Basically, for implementation of the method one requires two elongated structures that cross. The process can be achieved using materials regularly available in an X-ray or laser laboratory such as crossed wires of high-*Z* materials [Fig. 1 ▸(*c*)]. This approach has been tested in our experimental run and worked reasonably well. In this case, the polycapillary was inserted gradually, and aligned step-by-step. The precision of such pre-alignment is limited by the curvature of the elongated structures in the cross.

In the experiment preparation phase, care should be taken that the holder mounted on the motorized stage maintains its position within <0.5° in all directions when slits/wires are removed and the polycapillary is fixed with screws. In addition, the slit pockets of the holder [Fig. 1 ▸(*d*)] should fix the slits securely but allow for smooth aperture extraction.

## 3. Summary and outlook   

We demonstrate a fast, reliable and repeatable strategy of polycapillary alignment using two perpendicular masks imaged by a CCD camera to efficiently focus X-rays generated by a 4π polychromatic water-jet PXS. The pre-alignment technique is optimally used with special X-ray slits, but is also efficient when used with at hand laboratory materials such as wires. The method described in this communication is by no means specific to water-jet-based PXS but can be used equally well for different types of spherically radiating sources. Further development would be to employ a 3D-printed tube that either separates the two slits and fits them into pockets, or holds two tightly tensioned wires [Fig. 1 ▸(*d*)], creating a cross to enable a more precise pre-alignment of the *x* and *y* positions as well as the two rocking angles. After inserting the polycapillary optics into the alignment tube, the central capillaries are aligned, and only the position along the focal direction needs to be separately adjusted. The optimization problem is thus reduced from five independent undetermined alignment parameters to four coarsely aligned (*i.e.* determined) and one undetermined parameter. As the acceptance angle of the wires is 30° without requiring a pre-alignment of the optics, the use of a 3D-printed shape similar to the X-ray optics permits the fast and simple exchange of the alignment tool with the optics, reducing the experimental overhead of the procedure.

The greatest efficiency of polycapillary alignment in terms of optimized photon intensity and reduced time consumption can be achieved by combining the method demonstrated in this paper with automatic scanning procedures of photon intensities as a function of a polycapillary position and angle, similar to the automated test system for measuring X-ray transmission reported in the work by Rath *et al.* (1994 ▸). This alignment protocol will be particularly useful at user facilities, where preparation and measurement time is very limited, and users can be expected to request different X-ray beam parameters requiring frequent changes of focusing optics, but will be appreciated also in regular laboratories. In our case, the setup is being built for users of the ELI Beamlines facility. Thus, we foresee the need to use the developed tool on a regular basis due to frequent changes to the sample environment, which makes either removing or exchanging the optics necessary as well as misaligning the optics by accident quite likely. In particular, a central feature that will be offered to users is a wide range of possible pump/activating pulses, and a variety of sample delivery systems. The capability for rapid and flexible alignment will be a valuable and important time-saving feature of the instrumentation.
